# Laboratory Experiments and Grain Based Discrete Element Numerical Simulations Investigating the Thermo-Mechanical Behaviour of Sandstone

**DOI:** 10.1007/s10706-021-01794-z

**Published:** 2021-04-01

**Authors:** James Woodman, Audrey Ougier-Simonin, Anastasios Stavrou, Ioannis Vazaios, William Murphy, Mark E. Thomas, Helen J. Reeves

**Affiliations:** 1 University of Leeds, School of Earth & Environment, West Yorkshire, UK; 2 Present Address: Jacobs Engineering Group Inc., 1 City Walk, Leeds, UK; 3 British Geological Survey, Environmental Science Centre, Keyworth, UK; 4 Ove Arup & Partners Ltd, 13 Fitzroy Street, London, UK

**Keywords:** Thermo-mechanical, Micro-crack, Triaxial, Grain based DEM

## Abstract

Thermo-mechanical loading can occur in numerous engineering geological environments, from both natural and anthropogenic sources. Different minerals and micro-defects in rock cause heterogeneity at a grain scale, affecting the mechanical and thermal properties of the material. Changes in strength and stiffness can occur from exposure to elevated temperatures, with the accumulation of localised stresses resulting in thermally induced micro-cracking within the rock. In this study we investigated thermal micro-cracking at a grain scale through both laboratory experiments and their numerical simulations. We performed laboratory triaxial experiments on specimens of fine-grained sandstone at a confining pressure of 5 MPa and room temperature (20$^{\circ }\hbox {C}$), as well as heating to 50$^{\circ }\hbox {C}$, 75$^{\circ }\hbox {C}$ and 100$^{\circ }\hbox {C}$ prior to mechanical loading. The laboratory experiments were then replicated using discrete element method simulations. The geometry and granular structure of the sandstone was replicated using a Voronoi tessellation scheme to produce a grain based model. Strength and stiffness properties of the Voronoi contacts were calibrated to the laboratory specimens. Grain scale thermal properties were applied to the grain based models according to mineral percentages obtained from quantitative X-ray diffraction analysis on laboratory specimens. Thermo-mechanically coupled modelling was then undertaken to reproduce the thermal loading rates used in the laboratory, before applying a mechanical load in the models until failure. Laboratory results show a reduction of up to 15% peak strength with increasing thermal loading between room temperature and 100$^{\circ }\hbox {C}$, and micro-structural analysis shows the development of thermally induced micro-cracking in laboratory specimens. The mechanical numerical simulations calibrate well with the laboratory results, and introducing coupled thermal loading to the simulations shows the development of localised stresses within the models, leading to the formation of thermally induced micro-cracks and strength reduction upon mechanical loading.

## Introduction

Coupled thermo-mechanical processes present engineering challenges in a number of geological environments. Climate and weather can induce thermo-mechanical loading in surface rock, with diurnal temperature fluctuations causing strength and stability issues due to progressive damage (Lamp et al. 2017; Collins and Stock 2016). Thermal loading due to the geothermal gradient is paramount in deep mining, for instance the TauTona gold mine currently operates at a depth of 3.9 km under average temperatures of 60$^{\circ }\hbox {C}$ (Neingo and Tholana 2016), as well as deep tunnelling with rock temperatures of 46$^{\circ }\hbox {C}$ at the Gotthard Base Tunnel situated at a depth of 2.5 km (AlpTransit 2016; Rybach and Pfister 1994). Additionally, the introduction of anthropogenic heat sources, such as the heat generated by radioactive decay of high-level radioactive waste in geological environments, is projected to induce local thermal loading of approximately 60$^{\circ }\hbox {C}$ in addition to the natural geothermal gradient, peaking approximately 50 years after emplacement and decreasing thereafter (Quintessa 2012).

Research into the thermo-mechanical loading of rock began in the 1970s, with studies focussed on the Earth’s crustal behaviour. These early studies primarily investigated crustal lithologies such as granite, gabbro, dolerite and rhyolite and found that thermally loading specimens from room temperature up to temperatures as high as 800$^{\circ }\hbox {C}$ induced micro-cracking and caused irrecoverable thermal expansion. This thermal micro-cracking was attributed to result from localised stress concentrations induced by the thermal expansion heterogeneity of adjacent minerals with different thermal expansion coefficients, and occurred in the absence of a thermal gradient across a rock specimen (Richter and Simmons 1977). Subsequently thermal micro-cracking was shown to occur under confining pressures (up to at least 600 MPa) (Wong and Brace 1979; Heard and Page 1982; Bauer and Handin 1983). Adding mechanical and physical measurements to testing protocols showed that thermally induced micro-cracking was found to be significant enough to affect Young’s modulus (Simmons and Cooper 1978; Bruner 1979; Heard and Page 1982), ultrasonic velocities (Johnson et al. 1978), seismic attenuation (Johnson et al. 1978; Clark et al. 1981), fracture toughness (Meredith and Atkinson 1985), permeability (Bauer and Johnson 1979) and produce acoustic emissions (Johnson et al. 1978; Chen and Wang 1980). In comparison to studies focussed on crustal processes, there are fewer studies exploring thermo-mechanical effects at temperatures and pressures applicable to geo-engineering environments. Multiple large scale in-situ heater experiments have been undertaken to examine the effects of thermo-mechanical loading on rock masses (Barton 2007). But little work examining the small scale processes underpinning any macroscopic damage related to rock engineering practices exist. Recently Siegesmund et al. (2018) studied the thermal expansion of 65 granitic lithologies up to 100$^{\circ }\hbox {C}$, with implications for the use of granitoids as building stones and façade materials. Plevová et al. (2011) and Zhou et al. (2016) both undertook thermal studies on different sandstones. All three of these recent studies have shown that progressive damage can occur due to thermal loading at temperatures applicable to rock engineering environments.

Over the last 30 years, there have been significant advancements in the ability of different discrete modelling techniques to simulate the progressive damage, and micro-mechanical processes that occur during brittle rock failure. For a comprehensive review of the development and capabilities of different discrete modelling techniques we refer the reader to Lisjak and Grasselli (2014). In traditional Discrete Element Method (DEM) simulations, rock failure is captured through plastic yielding of rock blocks, or displacement on pre-existing discontinuities, and the creation of new fractures through brittle fracturing of existing blocks is not possible. Grain based modelling (GBM) is one technique to overcome this. The use of discs (or spheres in 3D) with cohesive bonds, which can break to simulate fracturing, and then allow fracture propagation through coalesence of bond breakage, has arguably become the most popular method of grain based modelling. Instead of discs or spheres, the Voronoi tessellation technique (Lorig and Cundall 1989) is also well accepted for simulating the microstructure of materials. The crystalline or granular micro-structure of rock is better represented by Voronoi tessellations due to full contact between grains and better interlocking due to the polygonal shapes. Micro-cracks develop when the stress level at Voronoi block contacts exceed a predefined threshold. In simulations utilising discs, the applied forces translate into tensile stresses at the contacts between discs and rotational moments, whilst in Voronoi simulations the forces can be resolved into tensile and shearing forces on the block boundaries. This is not fundamental when micro-cracks start to form, as micro-cracking in brittle rocks is tension dominated. However, with increasing deviatoric stress, and as yield starts to occur and micro-cracks accumulate and coalesce, shearing becomes dominant (Diederichs 2007), making Voronoi GBMs advantageous for simulating brittle fracturing. Many studies have shown the ability of these GBM methods to capture the progressive fracturing and failure behaviour of different rock types due to mechanical loading over a range of stresses within the brittle field (Christianson et al. 2006; Gao and Stead 2014; Damjanac et al. 2007; Stavrou and Murphy 2018; Farahmand 2017).

DEM GBMs have also proven to be a useful tool to scale the mechanical behaviour of intact rock at the laboratory scale to the behaviour of rock blocks at an engineering scale (Stavrou and Murphy 2018; Stavrou et al. 2019). The effects of thermo-mechanical loading have also been demonstrated in GBMs. Lan et al. (2013) simulated a large scale in-situ thermal loading experiment (the Äspö Pillar Stability Experiment (Andersson 2007; Andersson et al. 2009)), using a thermo-mechanically coupled tunnel scale Voronoi based GBM to capture thermo-mechanically induced displacements and provided new insights to the localised stress distribution and micro-cracking in a thermo-mechanically loaded rock mass. Park et al. (2015) created laboratory scale thermo-mechanically coupled Voronoi GBM simulations of the Hwangdeung granite, again showing the development of displacement due to thermal expansion and the formation of thermally induced micro-cracking, however no laboratory data were provided to allow comparison of the strength and deformation properties at elevated temperatures.

In this study, we undertake elevated temperature laboratory triaxial tests to investigate induced thermo-mechanical effects on sandstone microstructure and brittle failure micromechanisms. Experiments were conducted at a confining pressure of 5 MPa and temperatures ranging from 20 to 100$^{\circ }\hbox {C}$ to represent conditions for deep geo-engineering environments. We then further explore the progressive damage processes through thermo-mechanically coupled numerical simulations using DEM GBMs to represent the microstructure of the tested laboratory rock specimens to provide the first simulations of such thermo-mechanical experiments.

## Laboratory Testing

### Laboratory Specimens and Methods

Specimens used in this study were of Thornhill Rock, a light brown fine-grained sandstone from the Pennine Middle Coal Measures Formation (Lower Carboniferous), in West Yorkshire, UK (Stone et al. 2010). Blocks of dimension stone were obtained from Britannia Quarry, Leeds (53$^{\circ }$43’55” N 1$^{\circ }$35’49” W). Basic mechanical properties of the Thornhill Rock from this location have previously been characterised through laboratory testing and are summarised in Table 1. Eight specimens from one block of dimension stone were prepared in accordance with International Society for Rock Mechanics (ISRM) suggested methods (Ulusay 2014). Specimens were cored perpendicular to bedding with a diamond impregnated coring drill to 54 mm diameter and sawn to approximately 120 mm in length. Both ends were then ground flat to an accuracy of ±0.02 mm, not departing from perpendicularity to the axis of the specimen by more than 0.025 mm in 25 mm using a diamond sintered grinding wheel, thus preventing an uneven disctribution of stress on the specimen ends during experimentation (Hawkes and Mellor 1970). Specimens were oven-dried at 40$^{\circ }\hbox {C}$ for at least 48 h prior to testing.

**Table 1:** Properties obtained from baseline characterisation testing of the Thornhill Rock (Woodman et al. 2018; Woodman 2020). The standard deviation is the measure of variability reported with the mean values

Property	Unit	Mean value
Density ($\rho$)	${\hbox {kg m}^{-3}}$	$2250.0 \pm 22.12$
Porosity ($\varphi$)	%	$15.5 \pm 0.02$
Unconfined compressive strength (UCS)	MPa	$50.80 \pm 8.20$
Young’s modulus (*E*)	GPa	$8.70 \pm 1.26$
Poisson’s ratio ($\nu$)	−	$0.22 \pm 0.02$
Tensile strength ($\sigma _t$)	MPa	$4.20 \pm 0.60$
Cohesion (*c*)	MPa	9.63
Friction angle ($\phi$)	$^{\circ }$	49.73

Elevated temperature triaxial testing was undertaken at the Rock Mechanics and Physics Laboratory, British Geological Survey (BGS). Test procedures were designed to replicate thermo-mechanical boundary conditions that may be expected in a deep rock engineering scenario, such as a deep tunnel, mine or generic geological disposal facility with the emplacement of heat generating radioactive waste. The worst case thermal load on the rock mass in any of these cases is likely to be approximately 100$^{\circ }\hbox {C}$. Tests were therefore undertaken at ambient room temperature ($\approx$ 20$^{\circ }\hbox {C}$), 50$^{\circ }\hbox {C}$, 75$^{\circ }\hbox {C}$ and 100$^{\circ }\hbox {C}$, with two specimens tested at each temperature. All tests were undertaken at 5 MPa confining pressure. Depending on the density of overburden, ground saturation, the regional stress field and the coefficient of lateral earth pressure, this equates to a depth of between 200 and 500 m depth.

**Fig. 1:**
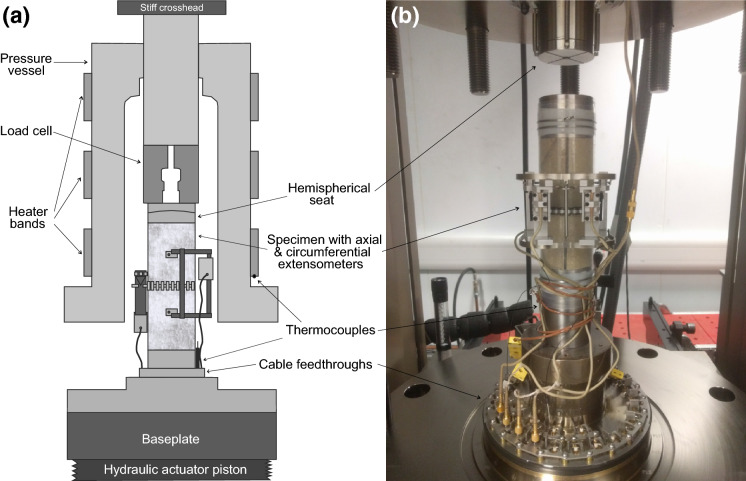
**a** Schematic diagram of an instrumented specimen set up in the pressure vessel for triaxial testing. **b** Photograph of a specimen set up in the pressure vessel for triaxial testing

The test apparatus consisted of a servo-controlled stiff frame (maximum axial load up to 4600 kN), with a confining pressure vessel (capable of pressure up to 140 MPa). The confining cell was fitted with external heater bands and utilised cascade control from internal and external thermocouples (accurate to ± 0.5$^{\circ }\hbox {C}$) (Fig. 1). A 1500 kN capacity force transducer (accurate to 0.32% of load) was used to measure the axial load. After preparation, the specimens were placed between two hardened steel platens encased in two layers of 0.5 mm Polytetrafluoroethylene (PTFE) heat shrink tubing and sealed using self-amalgamating silicone tape and stainless steel locking wires to prevent the ingress of confining fluid. The specimens were instrumented with two axial extensometers (accurate to ± 0.01%), positioned diametrically opposite each other, and a circumferential chain extensometer (accurate to ± 0.01%) positioned mid-length. A spherical seated platen was used between the specimen and the capacity force transducer to prevent eccentric loading. Finally a thermocouple was positioned inside the cell as close to the specimen as possible.

An initial axial pre-load of 2.3 kN was applied to specimens, to ensure a stable contact and alignment of the platens. In tests undertaken above ambient room temperature a confining pre-load of 0.5 MPa was then applied whilst heating was undertaken. Heating was undertaken at 1.5$^{\circ }\hbox {C min}^{-1}$ to the desired thermal load of either 50$^{\circ }\hbox {C}$, 75$^{\circ }\hbox {C}$ or 100$^{\circ }\hbox {C}$. A loading rate of 1.5$^{\circ }\hbox {C min}^{-1}$ ensured specimens were not thermally shocked, and ensured a low temperature gradient across the specimens. Slight temperature overshoot occurred in tests at 50$^{\circ }\hbox {C}$ and 75$^{\circ }\hbox {C}$, due to the temperature cascade control being calibrated at 100$^{\circ }\hbox {C}$ and fluctuations in room temperature. Based on a thermal conductivity (*k*) of 2.3 $\hbox {W m}^{-1}\hbox { K}^{-1}$, density ($\rho$) of 2250 ${\hbox {kg m}^{-3}}$ and specific heat capacity ($c_p$) of 700 ${\hbox {J g}^{-1}\hbox { K}^{-1}}$, thermal diffusivity ($\kappa$) can be calculated as:

1 $$\begin{aligned} \kappa = \frac{k}{\rho \cdot c_p} \end{aligned}$$

Thermal diffusivity of specimens is given as approximately $1.4\times 10^{-6}\hbox { m}^{2}\hbox { s}^{-1}$, and specimen radius (*r*) is $2.7\times 10^{-2}\hbox { m}$, therefore the time constant for temperature equilibrium ($r^2/\kappa$) (Wang et al. 2013), is approximately 900 s. To ensure thermal equilibrium across the specimen, temperature and pre-loads were held constant for 30 min prior to loading the specimen in axial extensometer control at an axial strain rate of $5\times 10^{-6}{\hbox { s}^{-1}}$. When the specimen began to yield and dilate volumetrically, control was switched to circumferential extensometer control at ${1\times 10^{-3}}{\hbox {mm s}^{-1}}$ to better control the radial dilation of the specimen and capture the failure process. After the specimen failed and residual strength was reached, the test was terminated at 2$\hbox { mm}$ circumferential extension.

Micro-structural analysis was also undertaken on laboratory specimens. Polished thin sections were prepared from specimens after thermo-mechanical loading, however the mechanical deformation was seen to overprint any thermal micro-cracking. Therefore two new specimens were prepared. One specimen (# 601) was prepared and oven dried at 40$^{\circ }\hbox {C}$ for 48$\hbox { h}$ as with specimens prepared for triaxial testing. The second specimen (# 602) was prepared and oven dried at 40$^{\circ }\hbox {C}$ for 48$\hbox { h}$, before being thermally loaded in a convection oven at 1$^{\circ }\hbox {C min}^{-1}$ to 100$^{\circ }\hbox {C}$, and held for one hour before being left to passively cool. Specimens were epoxy impregnated under vacuum to preserve deformation prior to polished blocks being made suitable for micro-structural analysis. Scanning Electron Microscopy (SEM), consisting of back scattered electron (BSE) and SEM-Cathodoluminescence (SEM-CL) imaging was undertaken using an FEI Quanta 650 Field Emission Gun SEM operated at 20$\hbox { kV}$.

### Laboratory Results

**Fig. 2:**
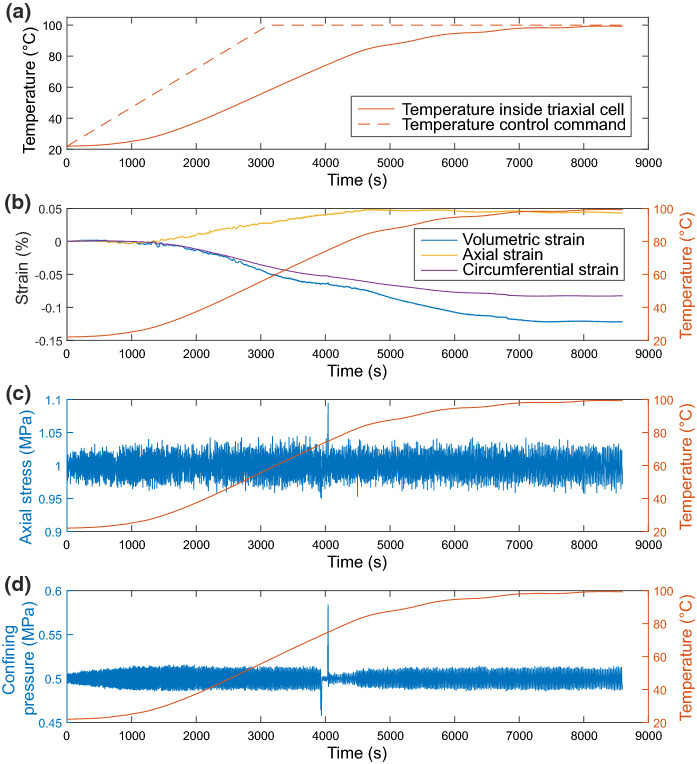
**a** Thermocouple data on the heating of Specimen # 562 within the triaxial pressure vessel, compared with the 1.5$^{\circ }\hbox {C min}^{-1}$ heating command. **b** Volumetric, axial and circumferential deformation of the specimen during heating due to thermal expansion. **c** Constant axial stress of 1$\hbox { MPa}$ maintained during thermal loading. **d** Constant confining pressure of 0.5$\hbox { MPa}$ maintained during thermal loading

Results of the eight thermo-mechanical triaxial tests undertaken in this study are tabulated in Table 2. The raw data from each test were processed, separating the thermal and mechanical loading portions of each test. Fig. 2 shows data from the thermal loading phase of a test undertaken at 100$^{\circ }\hbox {C}$. For the thermal loading of all specimens, a lag is observed in the first 1000$\hbox { s}$ between the heating command at 1.5$^{\circ }\hbox {C min}^{-1}$ and the heating of the specimen measured from the thermocouple within the triaxial pressure vessel. This is due to the initial heating of the steel pressure vessel and mineral oil confining fluid prior to heating of the specimen itself. Heating then occurs at a slightly slower rate (1.1$^{\circ }\hbox {C min}^{-1}$) than the heating command (1.5$^{\circ }\hbox {C min}^{-1}$), before the servo-control feedback reduces the heating rate and it plateaus to the desired temperature. Due to the calibration of the feedback on the temperature servo-controller, all tests undertaken at 50$^{\circ }\hbox {C}$ overshot the target temperature and were actually undertaken in the region of 53$^{\circ }\hbox {C}$ to 56$^{\circ }\hbox {C}$. All thermally loaded tests above 50$^{\circ }\hbox {C}$ show a peak in the confining pressure and axial stress during heating ($\approx$ 4000$\hbox { s}$ on Fig. 2c & d) of approximately 0.1$\hbox { MPa}$, which is due to manual repriming of the confining pressure intensifier, necessary due to the thermal expansion of the confining oil. Fig. 2 shows specimen deformation due to thermal expansion up to 100$^{\circ }\hbox {C}$. The axial extensometers do not show expansion, and instead record compaction during the heating phase. This may be as a result of increased ductility of the PTFE jacket resulting in slipping of the specimen within the jacket, and slipping of the extensometers on the jacket. This same result occurred during calibration testing on aluminium dummy specimens (Woodman 2020). However, the circumferential strain recorded from the circumferential chain extensometer shows dilation throughout heating. Isotropic thermal expansion is therefore assumed and the circumferential extensometer deformation ($\varepsilon _c$) is used to calculate the linear thermal expansion coefficient ($\alpha _L$) for each specimen as:

2 $$\begin{aligned} \alpha _L = \frac{\varepsilon _c}{\varDelta T \pi } \end{aligned}$$

where $\varDelta T$ is the change in temperature.

**Table 2:** Results of thermo-mechanical triaxial testing on specimens of Thornhill Rock. ($E =$ Tangential Young’s modulus. $\nu =$ Poisson’s ratio. CI = Crack initiation. CD = Crack damage. $\alpha _L$ = Coefficient of linear thermal expansion)

ID	Temp.	Length	Diam.	Peak diff. stress	*E*	${\nu }$	CI	CD	${\alpha _L}$
	($^{\circ }\hbox {C}$)	(mm)	(mm)	($\sigma _1 - \sigma _3$) (MPa)	(GPa)		(MPa)	(MPa)	${10^{-6}}{\hbox { K}^{-1}}$
565	24.0	128.80	54.02	99.45	13.81	0.20	38.93	74.45	–
566	20.1	124.71	54.04	97.21	15.90	0.19	40.03	74.76	–
504	56.3	119.79	53.98	89.09	15.55	0.20	37.05	66.82	3.15
503	53.4	117.65	53.98	89.41	15.64	0.20	35.68	67.00	4.29
501	75.2	121.46	53.99	85.72	15.78	0.21	35.40	63.67	3.78
505	74.8	119.22	53.99	87.57	15.77	0.21	36.23	64.64	2.99
562	100.0	124.46	53.98	83.32	15.47	0.21	35.22	62.38	3.43
563	100.0	129.97	54.00	82.94	15.00	0.22	33.84	61.31	3.19

All specimens subjected to thermal loading under pre-load conditions of 1$\hbox { MPa}$ axial stress and 0.5$\hbox { MPa}$ confining pressure displayed similar deformation during thermal loading, with linear thermal expansion coefficients ranging from ${2.99\times 10^{-6}}\hbox { K}^{-1}$ to ${4.29\times 10^{-6}}\hbox { K}^{-1}$.

**Fig. 3:**
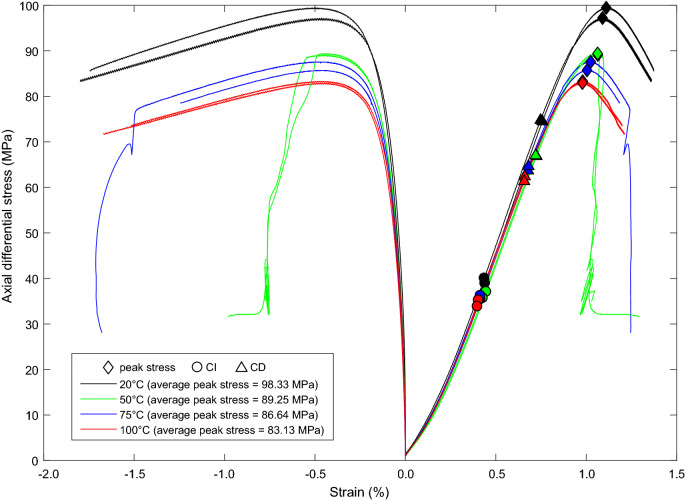
Axial stress versus radial and axial strain data for triaxial tests on specimens of Thornhill Rock, carried out at 5$\hbox { MPa}$ $\sigma _3$ and temperatures of 20$^{\circ }\hbox {C}$, 50$^{\circ }\hbox {C}$, 75$^{\circ }\hbox {C}$ and 100$^{\circ }\hbox {C}$. (CI = crack initiation. CD = crack damage)

The stress-strain curves (Fig. 3) for all temperatures display typical compressive brittle behaviour (Hoek and Bieniawski 1965). The repeat tests at each temperature show good repeatability, as expected from homogeneous specimens. Young’s modulus (*E*) (tangential modulus) and Poisson’s ratio ($\nu$) were calculated at 50% of the peak stress (Jaeger et al. 2007; Ulusay 2014) using measurements from the axial and circumferential extensometers. The crack damage (CD) threshold for each specimen was taken at the point of maximum volumetric compaction, and the crack initiation (CI) threshold was calculated using the lateral strain response method (Nicksiar and Martin 2012).

**Fig. 4:**
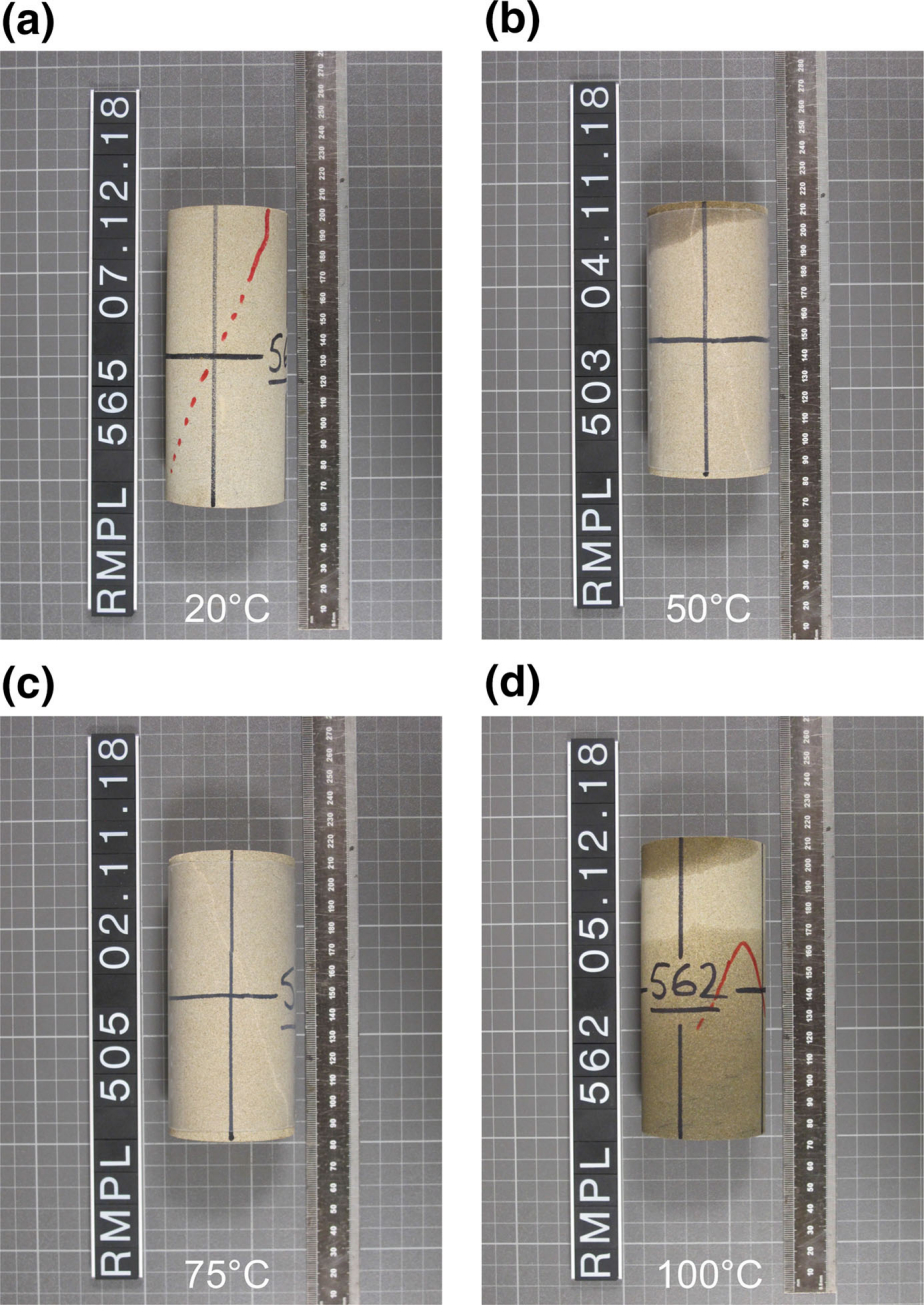
Photographs of specimens of Thornhill Rock after themo-mechanical triaxial testing, all tests were undertaken at 5$\hbox { MPa}$ $\sigma _3$. Note slight oil ingress to specimens **b** & **d**

A strength reduction is observed with increasing thermal loading. The peak differential strength reduces by approximately 17% between testing at room temperature and at 100$^{\circ }\hbox {C}$. Photographs of specimens after testing (Fig. 4) show the formation of a single shear plane fracture at all temperatures tested. The shear plane did not develop well in the tests undertaken at 100$^{\circ }\hbox {C}$, and the shear plane at 50$^{\circ }\hbox {C}$ was not distinctly different from tests undertaken at 20$^{\circ }\hbox {C}$ or 75$^{\circ }\hbox {C}$, despite the large stress drop post-peak. All developed fractures were planar, with little undulation or lobing and formed at angles between 18${^{\circ }}$ and 28${^{\circ }}$ from the vertical. It is important to note the ingress of oil to some specimens. This typically occurred at elevated temperatures as the PTFE jacket became more ductile, allowing ingress of oil to the specimen from the platens. However, the specimens were not saturated, suggesting the oil ingress occurred after the confining pressure had been removed during unloading. Consequently, this is not considered to affect the thermo-mechanical behaviour observed in the experiments.

## Grain Based Modelling

Following on from laboratory thermo-mechanical triaxial testing on specimens of Thornhill Rock, Universal Distinct Element Code (UDEC) v6.0 (Itasca 2014) was used to build Discrete Element Method (DEM) grain based models (GBM) representing the micro-structure of the laboratory specimens, allowing further investigation of the fundamental mechanisms of progressive damage under thermo-mechanical loads.

Conventionally in UDEC rock failure is captured either through plastic yielding of the rock matrix, or through sliding on explicitly modelled discontinuities (Lisjak and Grasselli 2014). This means that the formation of fractures through intact rock cannot be simulated. However, Lorig and Cundall (1989) introduced Voronoi tessellation to UDEC. The UDEC GBM utilises a Voronoi tessellation of deformable blocks to represent mineral grains, bonded at their contacts to represent the micro-structure of crystalline or granular rock. Fracture damage and development can therefore occur at the block boundaries when a stress level at the interface is exceeded either through thermal or mechanical loading (Lisjak and Grasselli 2014). The strength and stiffness of a GBM is governed by the grain contact micro-properties, as well as the grain size and grain size distribution of the Voronoi blocks, with the explicit generation, propagation and accumulation of micro-cracks. When a thermal or mechanical load is applied to the model, a perturbation is induced and a series of mechanical interactions between Voronoi blocks leads to the development and transmission of contact forces, the generation of localised heterogeneous stresses and eventually motion causing disturbance to the equilibrium of the system. The propagation speed of the movement depends on the physical micro-properties of the Voronoi elements within the discrete system. Further details on the equations of motion, conservation of momentum and energy equations, contact detection schemes, block deformability equations, and mechanical damping schemes for the 2D distinct element method utilised in UDEC can be found in Itasca (2014).

**Fig. 5:**
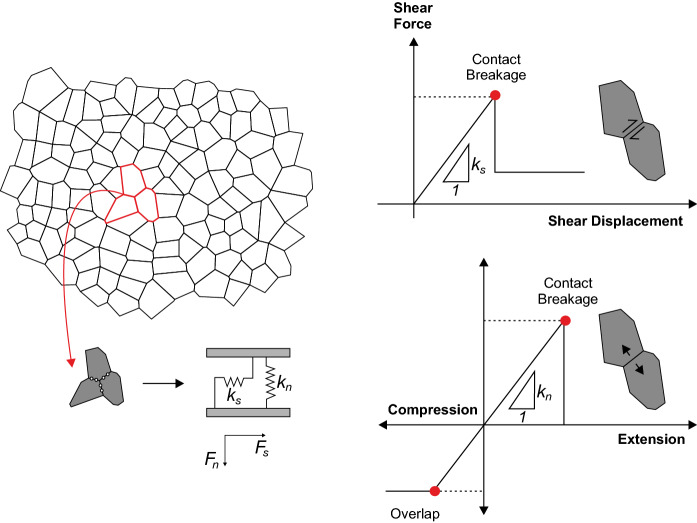
Voronoi structure and Mohr-Coulomb constitutive behaviour of the Voronoi contacts in the UDEC GBM. After Stavrou and Murphy (2018)

In all models in this study each Voronoi block is treated as an elastic continuum, sub-divided into triangular finite difference zones. Voronoi contacts obey a linearly elastic-perfectly plastic model, with normal and shear deformability represented by normal ($k_n$) and shear ($k_s$) stiffnesses (Fig. 5). The shear strength of the Voronoi contacts follow the Mohr-Coulomb criterion. In all simulations, if the induced contact forces exceed the tensile or shear strength of Voronoi contacts, a plasticity flag is set to declare the irreversible plastic state of the contact. Instantaneous softening then occurs with the micro-cohesion ($c_m$) and micro-tensile strength ($\sigma _{tm}$) of the Voronoi contact reduced to zero and the micro-friction angle ($\phi _m$) of the Voronoi contact reduced to a residual value. Forces are then redistributed allowing the relocalisation of stresses, which may in turn induce further micro-crack propagation and eventually macroscopic failure. This failure process allows the GBM to realistically capture the progressive failure process of micro-cracking in brittle crystalline and granular lithologies.

### Numerical Model Set Up

**Fig. 6:**
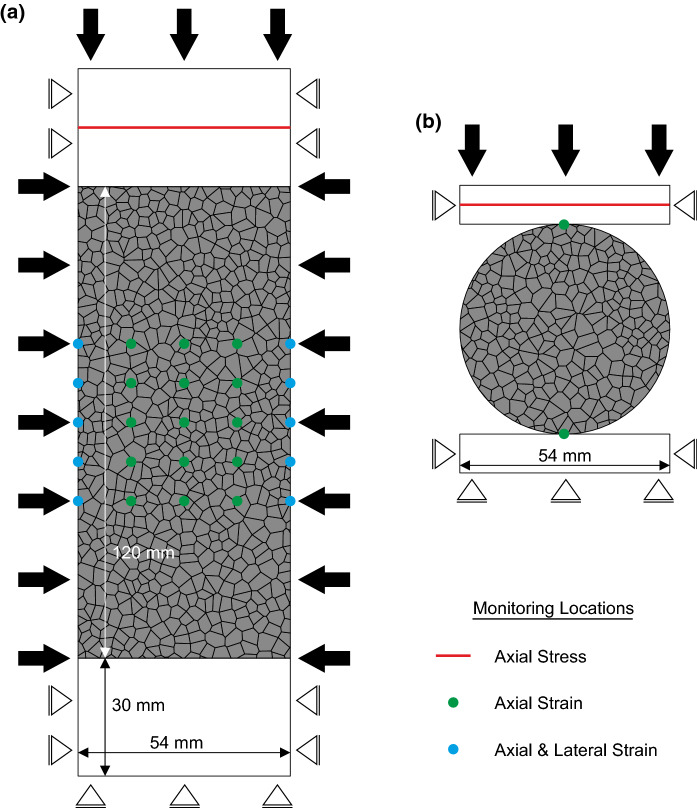
Model dimensions, boundary conditions and monitoring locations for numerical simulations. **a** Uniaxial and triaxial compression testing simulations. **b** Indirect tension Brazilian disc testing simulations

Specimens for numerical modelling were generated at the same scale as in the laboratory. Rectangular specimens (representing the cylindrical laboratory specimens in 2D plane strain) of $54 \times 120$ $\hbox { mm}$ were generated for uniaxial and triaxial compression testing, and 54$\hbox { mm}$ diameter circular specimens were generated for indirect tension Brazilian disc testing, for calibration purposes (Fig. 6). Both specimen types were bounded by steel platens top and bottom.

Specimens were discretised into Voronoi tessellations with an average grain edge length of 3$\hbox { mm}$. Different Voronoi block size and block size distributions are known to result in differing mechanical behaviour, as fractures can only form along contacts between adjacent Voronoi blocks, thus controlling the failure pattern. This phenomenon also results in models with a smaller block size having less effect on the failure pattern.

All GBMs have to make a compromise between grain size and computational efficiency. Hence, it is common for simulations to be run with a grain size considerably greater than the true grain size of the rocks being simulated e.g. (Christianson et al. 2006; Lan et al. 2013; Farahmand and Diederichs 2015), whilst ensuring that representative failure modes can be simulated. Gao and Stead (2014) found that a 160$\hbox { mm}$ tall model with a Voronoi block size of 4$\hbox { mm}$ block size allowed for representative failure mechanisms of both axial splitting and shear failure. Therefore despite the grain size in laboratory specimens of Thornhill Rock being in the range of 63 to 250 ${\upmu \hbox {m}}$, in this study a Voronoi block size of 3$\hbox { mm}$ is utilised to allow for representative failure mechanisms, whilst maintaining computational efficiency, and also satisfying the recommendation that the specimen diameter in uniaxial compression testing should be at least ten times greater than the size of the largest grain (Ulusay 2014). The Voronoi tessellation was developed with a relatively uniform grain size distribution to mimic the micro-structural homogeneity of the Thornhill Rock.

In uniaxial and triaxial compression testing simulations, the axial stress was measured using a *FISH* function at an imaginary crack within the upper platen. A grid of history points was created over the central 50$\hbox { mm}$ of the specimen (to simulate the area over which the axial and circumferential extensometers obtain measurements in the laboratory) and axial and radial strain measurements were monitored at these points (Fig. 6a). In indirect tension Brazilian disc simulations, the axial stress was monitored at an imaginary crack within the upper platen, and axial strain was calculated from the differing displacement between two monitoring points located at the top and bottom of the specimen. In addition, a *FISH* function was written to monitor the accumulation of tensile and shear micro-cracks during loading.

In the uniaxial and triaxial compression simulations, no pre-load was applied as is customary in laboratory experiments. The axial loading was applied as a constant velocity of 0.01$\hbox { m s}^{-1}$ in the *y*-direction at the upper platen whilst the lower platen was fixed in both the *x* and the *y*-directions. Although this is much faster than the rate that is applied in the laboratory (axial strain rate of ${5\times 10^{-6}} \hbox { s}^{-1}$), loading rate sensitivity analyses have shown that simulated strengths converge to consistent values when the loading rate is less than 0.25 $\hbox {m s}^{-1}$ (Tatone 2014; Mahabadi et al. 2010). In the case of triaxial compression testing, constant axial and confining pressures of equal magnitudes (i.e. hydrostatic stress) were applied at the specimens upper and lateral boundaries, and the models were taken to static equilibrium prior to the initiation of axial loading.

### Mechanical Model Calibration

The micro-parameters controlling the deformability (micro-Young’s modulus ($E_m$), micro-Poisson’s ratio ($\nu _m$), normal stiffness ($k_n$) and shear stiffness ($k_s$)) and strength (micro-cohesion ($c_m$), micro-friction angle ($\phi _m$) and tensile strength ($\sigma _{tm}$)) behaviour of the Voronoi micro-block assembly were estimated using a multi-stage parametric analysis in which the model response was calibrated against the deformability (*E*, $\nu$) and strength (*c*, $\phi$, $\sigma _t$) macro-mechanical properties of the Thornhill Rock. The iterative trial and error process approximately followed the procedures outlined by Christianson et al. (2006); Kazerani (2011); Gao and Stead (2014); Farahmand and Diederichs (2015) and, Stavrou and Murphy (2018). A series of unconfined compression tests, triaxial compression tests and indirect tensile Brazilian disc tests were carried out to calibrate the Voronoi contact and block micro-properties to produce macro-properties representative of the laboratory specimens.

**Fig. 7:**
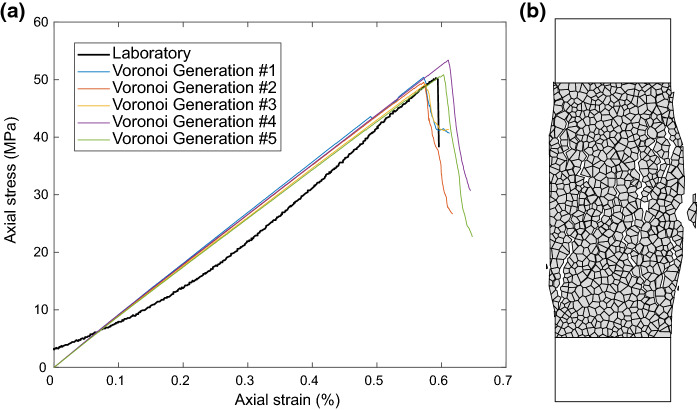
**a** Axial stress strain data from unconfined compression tests on five different Voronoi generations of UDEC GBM models calibrated to the properties of the Thornhill Rock, compared to a laboratory unconfined compression test on a specimen of Thornhill Rock.** b** UDEC GBM model after an unconfined compression test showing the formation of conjugate shear planes and axial splitting

A single set of micro-properties were calibrated and applied to all Voronoi blocks and contacts (Table 3). Previous studies (e.g. Farahmand and Diederichs (2015); Park et al. (2017)) have calibrated mineral-specific block and contact properties for polymineralic GBMs. However, as this study concentrates on the coupled thermo-mechanical behaviour of the simulations, and all micro-mechanical calibration involves non-unique solutions, we have assumed that a single set of calibrated mechanical properties is acceptable. A comparison between the mechanical properties obtained through laboratory characterisation testing and the calibrated macro-mechanical properties from numerical simulations are shown in Table 4. A good agreement is shown between all parameters, with the difference between laboratory and numerical simulation values being less than the sample variability seen in the laboratory (Table 1). To examine the repeatability of the mechanical behaviour five different random Voronoi tessellations were generated and tested, the results show good repeatability and prove the mechanical behaviour is independent of the generated Voronoi tessellation (Fig. 7).

**Table 3:** Calibrated micro-mechanical properties of the Voronoi blocks and contacts

Property	Units	Value
Elastic Properties of Voronoi Blocks		
Young’s modulus ($E_m$)	GPa	27.90
Poisson’s ratio ($\nu _m$)	-	0.22
Bulk modulus ($K_m$)	GPa	16.61
Shear modulus ($G_m$)	GPa	11.43
Elastic Properties of Voronoi Contacts		
Normal stiffness ($k_n$)	$\hbox { GPa m}^{-1}$	21235.36
Shear stiffness ($k_s$)	$\hbox { GPa m}^{-1}$	5308.84
Stiffness ratio ($k_n / k_s$)	–	4.00
Strength Properties of Voronoi Contacts		
Cohesion ($c_m$)	MPa	48.0
Friction angle ($\phi _m$)	$\circ$	27.0
Tensile strength ($\sigma _{tm}$)	MPa	8.0
Residual cohesion ($c_{mr}$)	MPa	0.0
Residual friction angle ($\phi _{mr}$)	$\circ$	15.0
Residual tensile strength ($\sigma _{tmr}$)	MPa	0.0

**Table 4:** Comparison of properties derived from laboratory characterisation testing and from calibrated mechanical UDEC GBM simulations

Property	Units	Laboratory	UDEC GBM	% Difference
Density ($\rho$)	$\hbox {kg m}^{-3}$	2250.00	2250.00	0.00%
Uniaxial compressive strength (UCS)	MPa	50.80	50.69	0.22%
Young’s modulus (*E*)	GPa	8.70	8.80	1.15%
Poisson’s ratio ($\nu$)	–	0.22	0.22	0.00%
Bulk modulus (*K*)	GPa	5.18	5.23	0.97%
Shear modulus (*G*)	GPa	3.57	3.61	1.12%
Tensile strength ($\sigma _t$)	MPa	4.20	4.22	0.48%
Cohesion (*c*)	MPa	9.63	9.99	3.74%
Friction angle ($\phi$)	$\circ$	49.73	48.00	3.48%

### Thermo-Mechanical Model Set Up

Following the successful mechanical calibration of Voronoi GBM simulations to the mechanical behaviour of the Thornhill Rock in laboratory characterisation testing, thermal properties were also added to the Voronoi GBM, and the simulations were thermo-mechanically coupled.

UDEC allows the simulation of transient heat conduction, and the development of thermally induced displacements and stresses. Using the explicit calculation scheme, at every thermal timestep, equations for conductive heat transfer (Fourier’s law) are solved numerically. Heat transfers across Voronoi block contacts without resistance, provided that the blocks are in contact. The thermal calculation procedure is coupled uni-directionally to the mechanical stress calculations through the linear thermal expansion coefficients applied to the Voronoi blocks (Itasca 2014). The confining fluid surrounding the specimen used to apply a confining pressure and thermal load during laboratory testing is not included in numerical simulations, therefore thermal loading was applied directly to the specimen and platens. The temperature for the start of all thermo-mechanical simulations was initialised at 20$^{\circ }\hbox {C}$. Thermal loading was then applied to the outer 1$\hbox { mm}$ of the specimen and platens in 1.5$^{\circ }\hbox {C}$ increments. A thermal timestep of ${3.0\times 10^{-5}}\hbox { s}$ was chosen and cycled for 3,000,000 thermal timesteps at every 1.5$^{\circ }\hbox {C}$ increment. Thermo-mechanical coupling was set to occur every 1,000 thermal timesteps and 100,000 mechanical timesteps (or when mechanical equilibrium was solved).

**Fig. 8:**
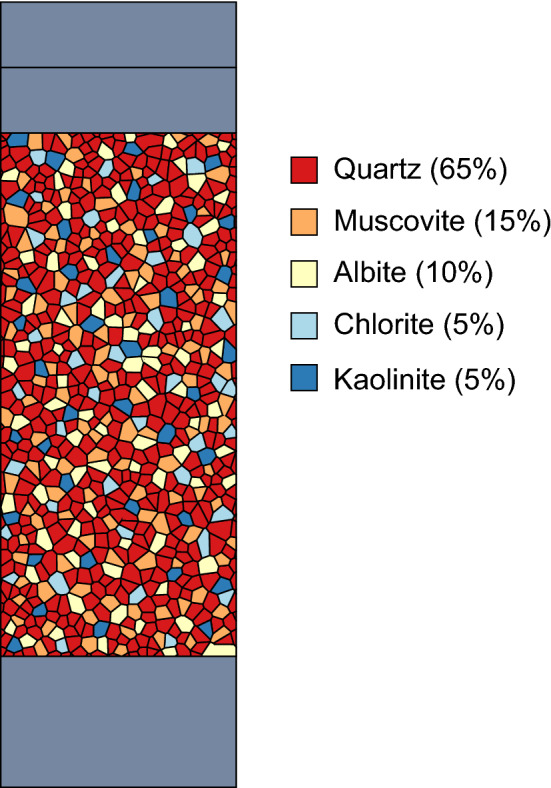
UDEC GBM with thermal material properties applied to Voronoi blocks in mineralogical percentages for the Thornhill Rock

Whilst only one set of mechanical properties were applied to all Voronoi blocks and contacts within the mechanical simulations, multiple different thermal properties corresponding to the mineralogy of the Thornhill Rock were applied in the thermo-mechanical Voronoi GBM simulations. This allows for heterogeneous thermal loading to occur within the specimen. Whole rock mineral percentages for the Thornhill Rock were obtained from quantitative X-ray diffraction (XRD) analysis and used to apply mineral percentages to the thermo-mechanical simulations (Table 5). The minor mineral constituents were not considered for thermal properties and the major mineral constituents were rounded to the nearest 5% for implementation of thermal properties. Thermal properties were randomly assigned to Voronoi blocks for different minerals in the relevant mineral percentages (Fig. 8).

**Table 5:** Thermal properties of minerals, and mineral percentages for Thornhill Rock used in thermo-mechanical simulations, obtained from quantitative XRD analysis

Mineral	Linear thermal expansion coefficient ($\alpha _L$) 10$^{-5}$ K$^{-1}$	Thermal conductivity (*k*) Wm$^{-1}$ K$^{-1}$	Specific heat capacity ($c_p$) J g$^{-1}$ K$^{-1}$	Thornhill Rock mineral percentage (%)
Quartz	1.60	7.69	698	65
Muscovite	1.16	2.32	760	15
Albite	0.54	2.31	709	10
Chlorite	0.90	5.14	600	5
Kaolinite	1.86	0.30	945	5

Numerical simulations were thermally loaded to the same temperatures as used in laboratory testing (of 50$^{\circ }\hbox {C}$, 75$^{\circ }\hbox {C}$ and 100$^{\circ }\hbox {C}$). Following the completion of coupled thermo-mechanical loading to the desired temperature, mechanical loading of the specimens was undertaken as in laboratory experiments. A confining pressure of 5$\hbox { MPa}$ was applied to the specimens and mechanical equilibrium was reached prior to initiating axial loading as in the triaxial mechanical calibration simulations.

### Thermo-Mechanical Simulation Results

**Fig. 9:**
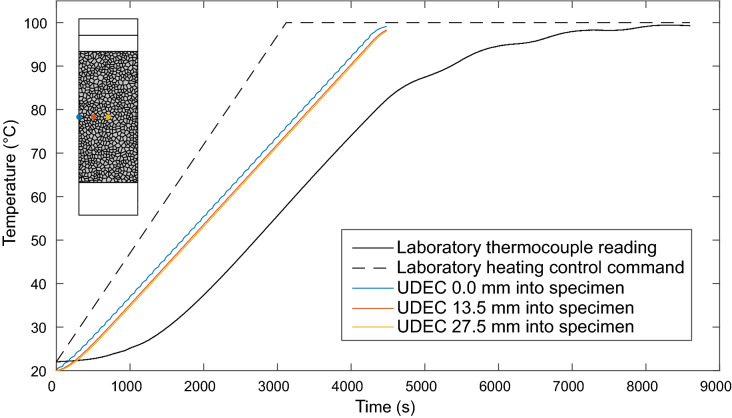
Monitoring of thermal loading throughout the model during numerical simulations compared with the thermal loading during laboratory experiments. Representative laboratory heating curve from a specimen tested at 100$^{\circ }\hbox {C}$ (Specimen # 562)

The selected thermal loading rate of 1.0$^{\circ }\hbox {C min}^{-1}$ in the numerical simulations was marginally lower than the observed thermal loading rate in the laboratory of $\approx$ 1.1$^{\circ }\hbox {C min}^{-1}$. However UDEC automatically over-rides and reduces the thermal timestep to maintain numerical stability dependent on the edge lengths of the Voronoi generation. This resulted in a slightly increased thermal loading rate. The temperature throughout each model was monitored at a grid of history points over the central 50$\hbox { mm}$ of the specimen (equal to the strain monitoring locations on Fig. 6). Fig. 9 shows the thermal loading in numerical simulations at three different places within the specimen compared to the heating command and thermocouple reading within the triaxial cell during a laboratory experiment. It is observed that the thermal gradient across specimens in the numerical simulations is less than 2$^{\circ }\hbox {C}$ and that, apart from the initial heating lag observed in the laboratory, the thermal loading rate in numerical simulations is concordant with the thermal loading rate in the laboratory.

The development of localised thermally induced stresses, and the propagation and accumulation of thermally induced micro-cracks was monitored during thermal loading. Micro-cracking was monitored by tracking the development of plasticity flags within the model (i.e. contacts reaching the irreversible plastic state as described by the constitutive model), and also the normal load present on those contacts, to deduce shear or tensile failure.

**Fig. 10:**
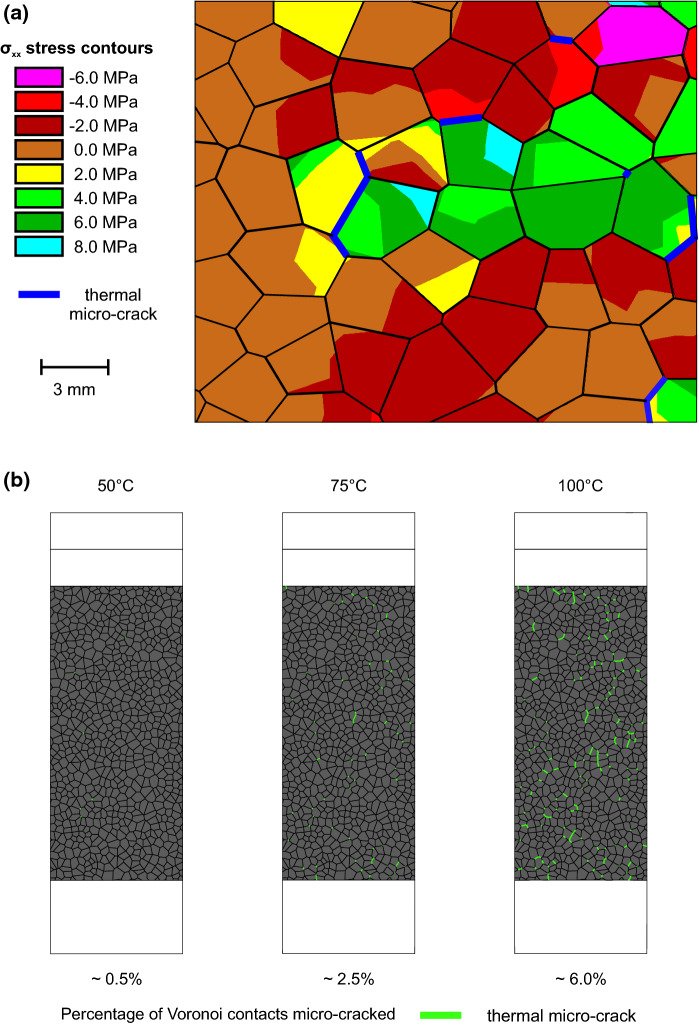
**a** A subset of a numerical simulation thermally loaded to 100$^{\circ }\hbox {C}$ showing *x*-component stresses ($\sigma _{xx}$). The left hand edge of the subset is the edge of the model. Negative stresses are compressive.** b** The amount of thermally induced micro-crack development in numerical simulations thermally loaded to 50$^{\circ }\hbox {C}$, 75$^{\circ }\hbox {C}$ and 100$^{\circ }\hbox {C}$

Fig. 10b shows the development of thermally induced micro-cracking within numerical simulations of the Thornhill Rock with increasing thermal loading. At 50$^{\circ }\hbox {C}$, only approximately 0.5% of Voronoi contacts within the model have cracked (approximately 25 Voronoi contacts out of a total of 5,000 contacts in the model). This increases to $\approx$ 2.5% of Voronoi contacts by 75$^{\circ }\hbox {C}$, and $\approx$ 6.0% of Voronoi contacts by 100$^{\circ }\hbox {C}$. The micro-cracks are seen to develop randomly throughout the specimen, with the exception of the outer 5$\hbox { mm}$ of the model on the vertical edges, where horizontal displacement is free to occur and thermally induced stresses do not develop. All thermally induced micro-cracks form in tension and appear to have no preferred orientation, although further cracking nucleates and propagates from already existing micro-cracks.

**Fig. 11:**
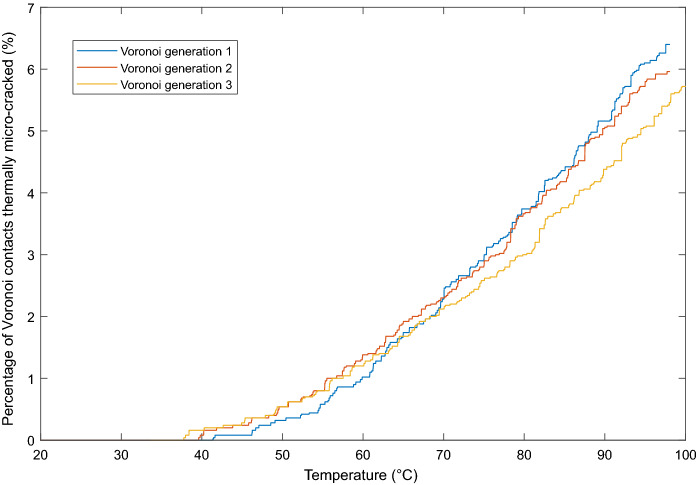
Percentage of thermally micro-cracked Voronoi contacts with increasing thermal loading. Simulations were run with three different random Voronoi generations

**Fig. 12:**
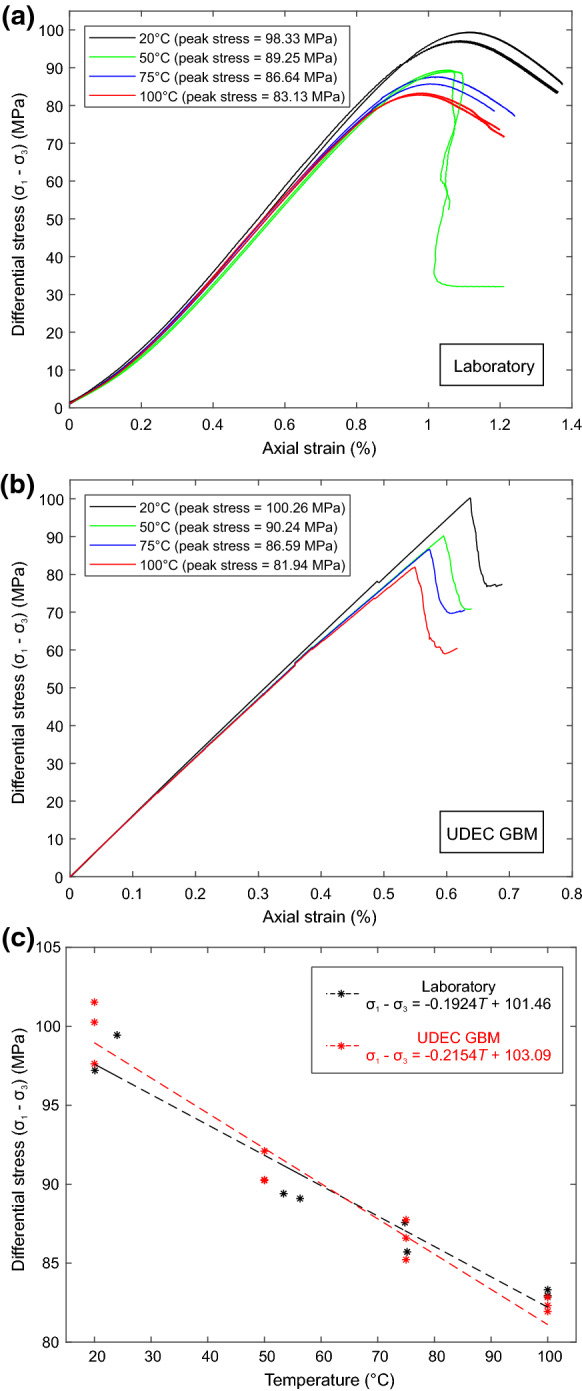
**a** Axial stress. vs. axial strain data for laboratory thermo-mechanical triaxial tests on intact specimens of Thornhill Rock. **b** Axial stress vs. axial strain data for UDEC GBM simulations of thermo-mechanical triaxial tests. **c** Comparison of peak strengths obtained for specimens subjected to different amounts of thermal loading in the laboratory and in numerical simulations

Micro-cracking is seen to initiate between 38$^{\circ }\hbox {C}$ and 42$^{\circ }\hbox {C}$ dependent on Vorronoi generation (Fig. 11). Micro-cracks accumulate slowly initially up to 60$^{\circ }\hbox {C}$, accumulating approximately linearly thereafter up to 100$^{\circ }\hbox {C}$. Upon mechanical loading after heating to 50$^{\circ }\hbox {C}$, 75$^{\circ }\hbox {C}$ and 100$^{\circ }\hbox {C}$, a reduction in peak strength is observed (Fig. 12b & c). No change is observed in Young’s modulus with increasing thermal loading, and it is not possible to apply the same methods for calculating the CI and CD thresholds as in the laboratory.

## Discussion

### Laboratory Testing

Our laboratory triaxial testing data show that increased thermal loading causes reduced strength in the studied Thornhill Rock (Fig. 12c), in line with experimental observations of strength reduction due to thermal loading in similar lithologies (Siegesmund et al. 2018; Plevová et al. 2011; Zhou et al. 2016; Zhang et al. 2013). The reduction in strength with thermal loading can be explained through the formation of thermally induced micro-cracks. The heterogeneous thermal expansion of the mineral grains is large enough to allow localised stresses to accumulate within the specimens, to a point at which inter-granular tensile micro-cracking occurs (Fredrich and Wong 1986). The CI and CD thresholds are also seen to reduce with increasing thermal loading, proportionally with the peak strength, confirming that the weakening is occurring prior to mechanical loading due to thermal micro-cracking during the thermal loading phase of the experiments. An increase in Poisson’s ratio is observed with increasing thermal loading, however, little change is observed in Young’s modulus. The increase in Poisson’s ratio must therefore occur due to increased radial deformation. The pre-load mechanical conditions applied during thermal loading were not hydrostatic. It is therefore likely that tensile micro-cracking will preferentially occur parallel to the axial load, causing increased radial deformation.

**Fig. 13:**
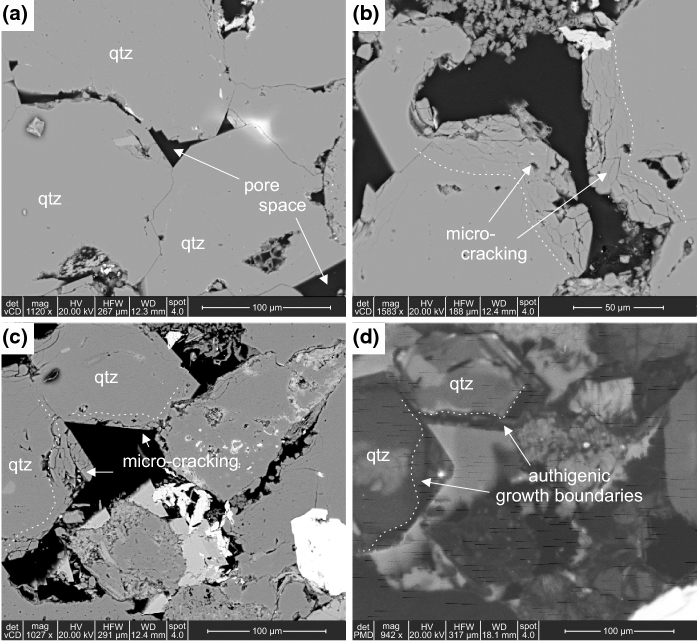
**a** BSE image of Specimen # 601 (oven dried at 40$^{\circ }\hbox {C}$).** b** BSE image of Specimen # 602 (thermally loaded to 100$^{\circ }\hbox {C}$).** c** BSE image of Specimen # 602 (thermally loaded to 100$^{\circ }\hbox {C}$).** d** SEM-CL image of Specimen #602 (the same view as BSE image shown in c))

Micro-structural analysis of specimens subjected to just thermal loading, further validate the development of thermally induced micro-cracking. The specimen not subjected to thermal loading except to drive off moisture (# 601) shows largely planar contacts between quartz grains (Fig. 13a). Minor inter-granular micro-cracking is observed between quartz grain contacts, and at boundaries between quartz grains and pore space, likely due to the specimen preparation. However, the specimen loaded to 100$^{\circ }\hbox {C}$ (# 602) shows considerable intra- and inter-granular micro-cracking (Fig. 13b & c). Intra-granular micro-cracking dominantly occurs at the edge of quartz grains, parallel to grain boundaries, and micro-cracks have increased apertures compared to those observed in Specimen # 601. Fig. 13d shows the same image as Fig. 13c in SEM-CL. Quartz grains are shown to have multiple phases under SEM-CL, with authigenic overgrowths into the pore space occurring on the detrital quartz. The intra-granular micro-cracking is largely limited within these overgrowths, and rarely propagates through the detrital grains, which may suggest heterogenous thermal and mechanical properties within the crystallographic structure of the quartz grains.

### Numerical Simulations

The thermo-mechanical triaxial simulations of the Thornhill Rock show comparable results to laboratory testing, with thermal loading alone causing progressive damage to specimens through the propagation and accumulation of tensile micro-cracks. Micro-cracking initiates at approximately 40$^{\circ }\hbox {C}$, and apart from the initial 15$^{\circ }\hbox {C}$ the micro-cracks accumulate linearly with increasing temperature up to 100$^{\circ }\hbox {C}$. This linear behaviour is expected, as whilst thermal properties vary with temperature (Sugawara 1968), this variation is typically very small over the temperature range applicable in this study, and therefore constant thermal properties were used in simulations. Fig. 10a shows a subset of the model contoured with the *x*-component of the stress tensor ($\sigma _{xx}$) after thermal loading to 100$^{\circ }\hbox {C}$. As expected, no horizontal stresses occur at the model boundary (left hand edge) as no confinement is yet applied, and horizontal displacement is possible as thermal expansion occurs. However, further within the model, it can be seen that localised stresses start to develop, with regions both in tension and compression, and the formation of micro-cracks in areas of tension, where the tensile strength ($\sigma _{tm} =$ 8.0$\hbox { MPa}$) of the Voronoi contacts is exceeded.

When assessing the mechanical strength of specimens after thermal loading, simulations of the Thornhill Rock show good agreement with the loss of strength observed in the laboratory, with the existing thermally induced micro-cracks acting as nucleation points for further progressive damage to occur (Fig. 12). Whilst the observed strength reduction is comparable, the numerical simulations show increased stiffness compared to the laboratory specimens. This is likely due to the Mohr-Coulomb constitutive models inability to simulate poro-elasticity and yielding, resulting in increased stiffness.

Overall, the thermo-mechanically coupled calibrated GBMs can capture micro-cracking as a mechanism of progressive damage, reproducing the stress-strain behaviour of laboratory specimens. Whilst good agreement is found between laboratory results and numerical simulations, the numerical simulations deform in 2D plane strain and therefore can not be used as predictive tools to quantify crack densities or distributions in the laboratory specimens. It is also accepted that the simulations are simplifications and neglect other potential mechanisms of progressive damage such as pore collapse and grain comminution. The simulations also do not account for specimen porosity, heterogeneous mechanical properties or anisotropic mechanical and thermal grain properties, which as shown by microstructural analysis of laboratory specimens are likely important factors in the progressive damage that occurs in laboratory specimens.

## Concluding Remarks

In this study we undertook elevated temperature laboratory triaxial testing on specimens of fine-grained sandstone. Increased thermal loading caused reduced strength upon mechanical loading, attributed to progressive damage through thermally induced tensile micro-cracking. Microstructural analysis showed the thermally induced micro-cracking to occur along quartz-quartz grain boundaries, and at boundaries between quartz grains and pore space, with micro-cracking dominantly occurring within authigenic overgrowths of the quartz grains. Thermo-mechanically coupled DEM GBM simulations were undertaken representing the microstructure of the laboratory specimens. Numerical simulations showed the onset and development of tensile thermal intra-granular micro-cracking due to the accumulation of localised thermal stresses from heterogeneous thermal properties of the polymineralic specimens. The thermal micro-cracking caused reduced strength with subsequent mechanical loading, with the strength reduction correlating well with laboratory results, showing the ability of a DEM GBM to help establish the mechanisms of damage and capture representative thermo-mechanical behaviour of brittle granular rock.

Interesting questions can be posed for future work regarding up-scaling the results of this study. It is widely recognised that the strength of intact rock decreases with increasing scale due to increased heterogeneity and the presence of critically orientated micro-defects (Hoek and Brown 1997; Tsur-Lavie and Denekamp 1982; Stavrou and Murphy 2018). However, whether the increased heterogeneity and micro-defects may also allow for increased thermally induced micro-cracking to occur, or whether micro-defects may inhibit the accumulation of localised thermally induced stresses is currently unknown. As well as introducing further heterogeneity and micro-defects to the numerical simulations, adding complexity to the simulation properties such as anisotropic grain fabrics (Ghazvinian et al. 2014) and anistropic grain properties (both mechanical and thermal), would provide further insight into the progressive damage of specimens.

## Declarations

### Availability of data and material

The laboratory data associated with this manuscript is archived on the National Geoscience Data Centre repository (10.5285/84abfa01-e81b-4d68-b6ee-e8f37e16cafd).
